# Practice, Experiences, and Facilitators of Simulation-Based Training During One Year of Implementation in 30 Hospitals in Tanzania

**DOI:** 10.1177/23779608241309447

**Published:** 2025-01-03

**Authors:** Benjamin A. Kamala, Robert Moshiro, Florence S. Kalabamu, Torgeirsen Kjetil, Godfrey Guga, Beatrice Githiri, Justine Samson, Philimon Chavala, Grace Qorro, Damas Kayera, Ivony Kamala, Catherine Massay, Paschal Mdoe, Vickfarajaeli Daudi, Esto Mduma, Shally Mwashemele, Felix Bundala, Hege Ersdal, Sara Rivenes Lafontan

**Affiliations:** 1 Department of Research, 380181 Haydom Lutheran Hospital, Manyara, Tanzania; 2 92976 Muhimbili University College of Health Sciences, Dar es Salaam, Tanzania; 3 108082 Hubert Kairuki Memorial University, Dar es Salaam, Tanzania; 4 542297 SAFER, Stavanger, Norway; 5 Faculty of Health Sciences, 621781 University of Stavanger, Stavanger, Norway; 6 108094 Kilimanjaro Christian Medical University College, Moshi, Tanzania; 7 214351 UNICEF, Dar es Salaam, Tanzania; 8 223217 Ministry of Health Community Development Gender Elderly Children, Dodoma, Tanzania; 9 Department of Simulation, 60496 Stavanger University Hospital, Stavanger, Norway; 10 Institute of Nursing and Health Promotion, 60499 Oslo Metropolitan University, Oslo, Norway

**Keywords:** simulation-based training, safer births, low dose, high frequency, facilitator-led team simulations, skills training, innovation

## Abstract

**Introduction:**

Enhancing the proficiency of healthcare workers (HCWs) in handling birth-related complications is crucial for reducing maternal and newborn morbidity and mortality. To achieve this, the Safer Births Bundle of Care offers a comprehensive set of innovative, simulation-based training interventions designed to strengthen the skills and competencies of HCWs working as skilled birth attendants.

**Objective:**

To describe the use of *in-situ* low-dose, high-frequency simulation-based training, and the experiences of this usage among HCWs and stakeholders at facilities in Tanzania.

**Methods:**

This mixed-methods study included quantitative and qualitative data collected between July 2021 and July 2022 across 30 health facilities in five regions of Tanzania. NeoNatalie Live (NNL) simulators were installed for independent skills and scenario training, and *in-situ* facilitator-led team simulations were introduced. The training frequency was analyzed using descriptive and analytical statistics, and mentorship and supervision reports were analyzed using qualitative content analysis.

**Results:**

A large and sustained number of *in-situ* NNL skill-training sessions (*n* = 35,101) and facilitator-led team simulations (*n* = 266) were conducted during the first year. Clinical burden per HCW did not affect the frequency of NNL skills training at the health facility level (*r* = −0.16, *p* = .40) nor facilitator-led team simulations. There was a positive but weak correlation between the frequency of facilitator-led team simulations and NNL skills training (*r* = 0.34, *p* = .05). Qualitative data showed a high degree of motivation and participation among all stakeholders, and active use of hospital data, both clinical indicators and training data, was perceived as a success factor.

**Conclusion:**

Facilitator-led *in-situ* simulation training was more likely to occur where individual skills-training sessions were recorded. Training sessions took place regardless of the increased workload.

## Introduction

The prevalence of maternal and newborn deaths in low- and middle-income countries (LMICs) is unacceptably high, despite coordinated global efforts (Paulson et al., 2021; World Health Organization, 2023). Most of these deaths are largely linked to suboptimal standards of care before, during, and after labor and delivery. In Tanzania, the neonatal mortality rate is estimated at 24 deaths per 1,000 live births (Tanzania Demographic and Health Survey and Malaria Indicator Survey 2022 Key Indicators, n.d.; UN Inter-agency Group for Child Mortality Estimation, n.d.). Moreover, data from the Tanzania Demographic Health Survey (2022) show a decline in under-five mortality, infant mortality, and postneonatal mortality but an increase in neonatal mortality, from 20 to 24 deaths per 1,000 live births between 2017 and 2022 (Sumankuuro et al., 2018).

Appropriate interventions to improve the quality of care around labor and birth are critical for averting preventable maternal and perinatal deaths (Jennings et al., 2019; Mduma et al., 2015a; Simonneau et al., 2013). These interventions include increasing the availability of skilled, competent, and motivated staff with the necessary tools to provide routine intrapartum care, to identify and manage intrapartum-related complications, and to provide immediate support for the resuscitation of newborns (Goldenberg et al., 2018; Zaka et al., 2018). This has not yet been achieved in Tanzania due to several compounding systemic challenges and inadequate training opportunities for capacity building (Ersdal et al., 2023; Hemming et al., 2015; Kamala et al., 2021).

## Review of Literature

There is evidence that *in-situ* low-dose, high-frequency (LDHF) training and a culture of continuous quality improvement (CQI) at the facility level are essential for retaining and translating skills from training into clinical practice in order to improve care and reduce morbidity and mortality (Gomez et al., 2018; Jennings et al., 2019; Mduma et al., 2015a). However, many LMICs, such as Tanzania, lack adequate tools, and this is combined with limited knowledge and skills about conducting LDHF training and CQI effectively.

To improve the quality of maternal and newborn care and strengthen skills in lifesaving interventions around birth, the Safer Births Bundle of Care (SBBC) was implemented by 30 hospitals in collaboration with the Ministry of Health (MoH) and the President's Office, regional administration and local government, and other local and international partners in Tanzania. The SBBC includes a package of proven innovative clinical and training tools in combination with *in-situ* LDHF simulation-based training and the use of local data to visualize gaps in clinical care (in line with national guidelines and standard operating procedures), thereby guiding the direction of training activities (i.e., tailor-made simulation-based training activities responding to the identified gaps (Kamala et al., 2021)). The recently published SBBC halfway evaluation describes a steady trend toward a reduction in early newborn and maternal mortality and a fluctuation of reported fresh stillbirths across times and regions (Ersdal et al., 2023).

### Objective of the Study

To describe the implementation of *in-situ* LDHF simulation-based training and the experiences of the SBBC bundle among healthcare workers (HCWs) and other stakeholders at health facilities in Tanzania.

## Methods

### Design

**Figure 1.**
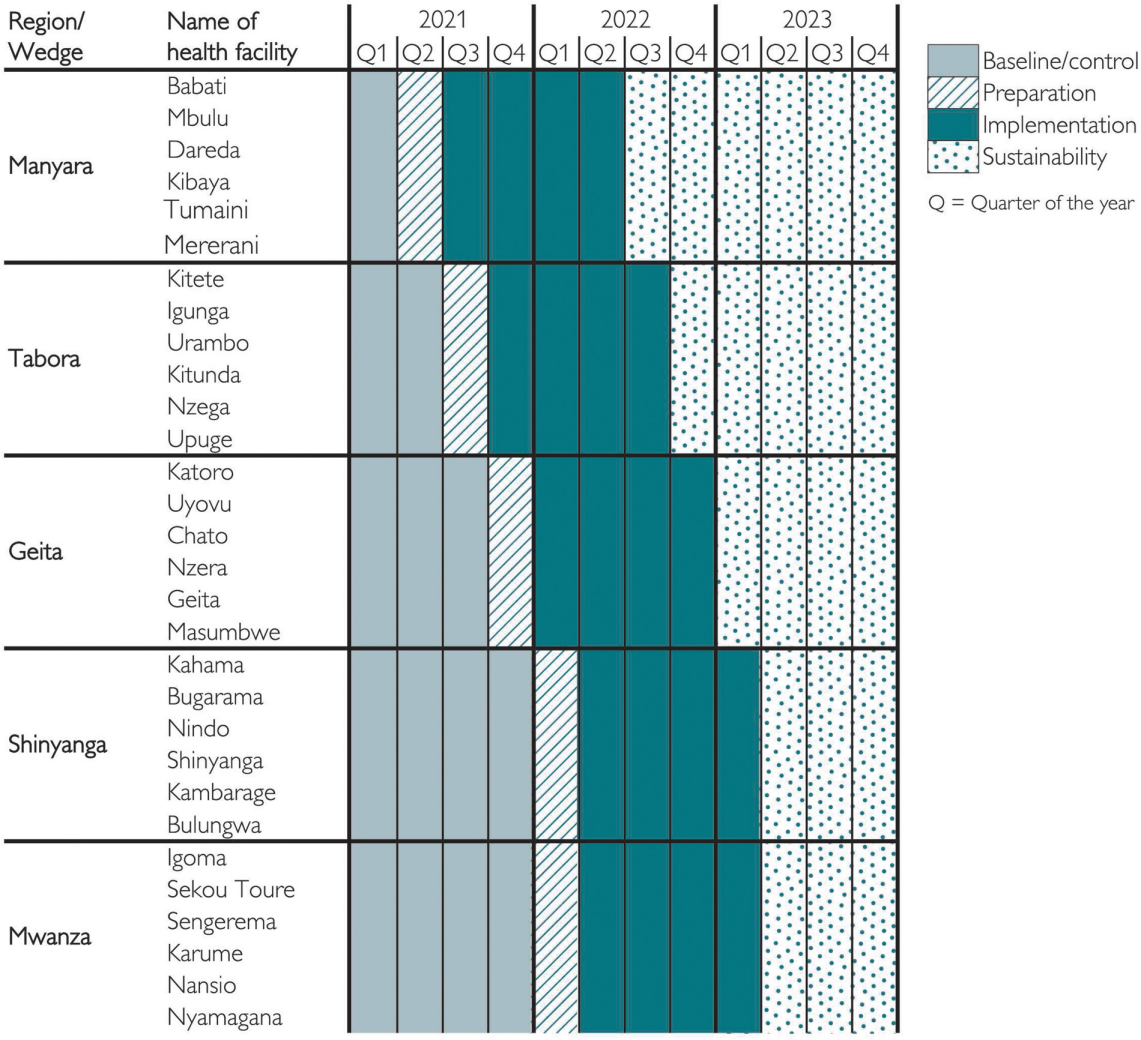
Periods for Interventions in the different regions (wedges) and health facilities (clusters) during the roll-out of the study.

The present study is a mixed-methods study that is embedded in the main SBBC implementation study and includes quantitative and qualitative SBBC data collected between July 2021 and July 2022. Figure 1 presents an overview of the regions, facilities, and periods for data collection for preimplementation (baseline) and postimplementation. The SBBC was implemented as a stepped-wedged cluster randomized implementation study (ISRCTN Registry: ISRCTN 30541755 dated 12/10/2020) (Hemming et al., 2014).

### The SBBC Intervention Description

**Figure 2.**
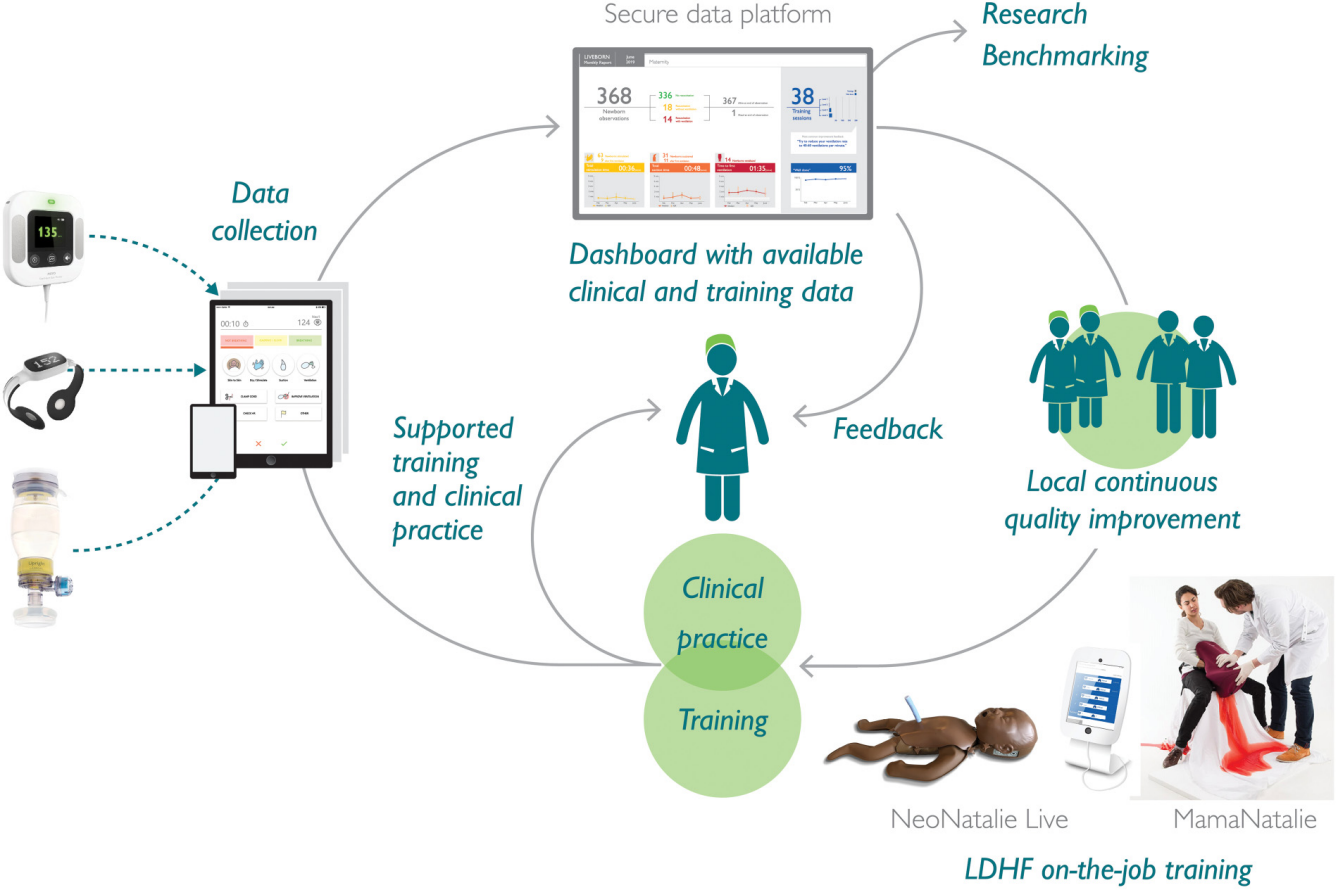
Integrating the innovative Safer Births Bundle of Care (SBBC) tools for clinical care, data collection and training to facilitate continuous quality improvement efforts. LDHF = low-dose, high-frequency.

The SBBC interventions target emergency obstetric and newborn care (EmONC)—that is, labor monitoring, preventing, and managing postpartum hemorrhage; managing difficult deliveries; and resuscitation of a nonbreathing newborn using systematic *in-situ* LDHF simulation-based training. The SBBC bundle includes innovative clinical tools—namely, the electronic fetal heart rate monitor (Moyo), the electronic newborn heart rate meter (NeoBeat), and the upright newborn bag mask (Figure 2). Safer Births Bundle of Care also includes training tools—namely, MamaNatalie and NeoNatalie Live (NNL) simulators (all equipment by Laerdal Global Health). These tools have been described in detail in a protocol publication (Kamala et al., 2021). The NNL newborn trainer enables short and flexible training on newborn ventilation and resuscitation (Chang et al., 2022; Haynes et al., 2021)—for individual and scenario training (collectively termed NNL skills training). The NNL is placed strategically within the labor wards, enabling HCWs to practice independently whenever possible.

In addition to NNL skills training, *in-situ* facilitator-led scenario team simulations to practice the management of complications during and after labor were introduced at all facilities. National facilitators and facility champions (see below) were responsible for organizing and facilitating this training regularly. A learning corner (a dedicated place where the training took place) was established within the labor ward of each facility. Most of the health facilities scheduled their own facilitator-led *in-situ* team simulations. All the innovative tools mentioned above are designed to facilitate LDHF simulation-based training coupled with local facility data to drive CQI using the plan-do-study-act cycle approach (Boaden, 2009). The training interventions target the improvement in the provision of EmONC. To enhance CQI through a gradual and sustainable local culture change through existing structures and employed personnel—that is, national facilitators and facility champions—were specifically trained to be responsible for local capacity building as part of the SBBC intervention.

#### Training of National Facilitators

**Figure 3.**
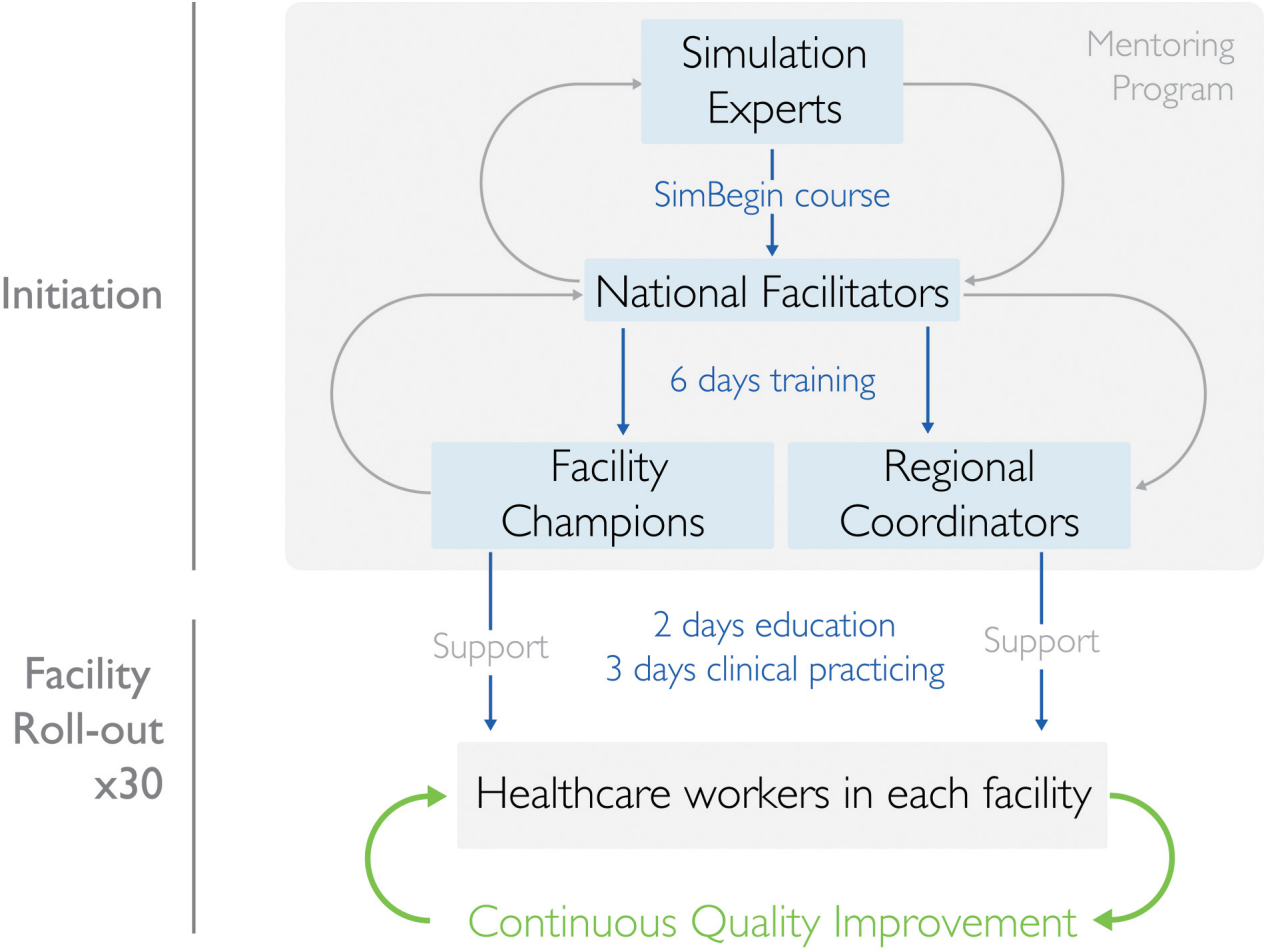
Illustration of the planned training cascade and implementation strategy.

Fifteen healthcare providers were chosen to participate in the two-week training that included one week of SimBegin. SimBegin (SimBegin, n.d.) is an introductory simulation-based training program for healthcare simulation. The program focuses on constructive facilitation, nontechnical skills, learners’ and patients’ safety, reflection-based debriefing models, different training modalities, simulation training for CQI, and mentoring of other simulation practitioners to help them improve their simulation skills. It entails giving HCWs time to reflect on their activities, learn to appreciate what is done well, and then discuss things they want to change and/or do better in the future (Hollnagel, 2015) (Figure 3).

#### Training of Facility Champions

In total, 72 champions, two per facility, and a labor ward charge nurse from each health facility were identified and trained for six days in three batches by the national facilitators (Figure 3). They were selected in collaboration with hospital management. They were supposed to have no administrative roles but to have the capacity to motivate peers to perform better and oversee *in-situ* training in their facility. Facility champions initiated, led, facilitated, and documented all *in-situ* SBBC training sessions. Additionally, the facility champions used local facility patients’ data (captured by dedicated data collectors) from weekly and monthly reports (Figure 2) to lead periodic debriefings (i.e., from their actual clinical performances), stimulating HCWs to reflect on their care.

#### Facility-Based HCWs’ Training

After the facility champions’ training, an onsite two-day training course and a three-day clinical mentorship, targeting all HCWs and service providers available at the facility maternity ward, postnatal ward, and newborn ward, were conducted (Figure 3). With support from the regional project coordinators, the facility champions assisted the national facilitators in training and mentorship.

#### Mentorship and Supportive Supervision Visits

For each region during the implementation of the SBBC, scheduled and ad hoc supportive supervision and mentorship visits were carried out. These were conducted by national facilitators collaborating with the SBBC national coordinator, regional coordinators, and regional health management teams. These scheduled mentorship and supportive supervision visits allowed for two-way communication to improve practice through skills and knowledge-sharing between mentors/supervisors and mentees/supervisees. For each mentorship visit, there were tailored objectives for each facility depending on previous visits and supported by different facility data collected from respective facilities from different sources.

### Setting

The study was implemented in 30 health facilities in five regions of Tanzania: Manyara, Tabora, Geita, Shinyanga, and Mwanza. All the facilities are categorized as comprehensive EmONC facilities. They represent different levels of the health system: regional referral hospitals (RRH), district hospitals (DH), and health centers (HC). The selection of regions and facilities was conducted in collaboration with the MoH and the President's Office, regional administration, and local government, based on the volume of deliveries and the burden of maternal, perinatal, and neonatal mortality. The study recruits all HCWs working in the labor ward and obstetric theater at the facilities included.

### Sample

The study's participants were HCWs from 30 sites in the five regions. All HCWs working in the labor ward in all 30 facilities were eligible for inclusion in the study. The number of midwives in each facility changed over time due to staff turnover.

### Data Collection

The protocol publication describes overall data collection and management (Kamala et al., 2021). For the current study, three types of data sources were analyzed and reported from July 2021 to July 2022 (Table 1).

**Table 1.** Data Sources, Content, and Type of Data.

Data source	Recorded diaries	NeoNatalie Live application	Mentorship and supervision reports
Content	Facilitator-led *in-situ* team simulation training	NeoNatalie Live skills and scenario training (NNL skills training)	Status during consecutive supervisions
Type of data	Quantitative	Quantitative	Qualitative

#### Facilitator-led *in-Situ* Team Simulation Training Data

Clinical diaries on facilitator-led *in-situ* team simulation training sessions were recorded in the counter books at each health facility and kept at learning corners. Data collected included facilitator-led team simulation training on postpartum hemorrhage management and newborn resuscitation. The diaries contained the objectives of the training, the type of scenario that the team had practiced (“helping babies breathe” or “helping mothers survive”), participants, what was done well during the scenario, challenges and areas that needed improvement, and action points or “take-home messages.” Data from the diaries were collected manually, scanned weekly, and sent to Haydom Lutheran Hospital, where it was entered into an electronic database, translated by dedicated personnel, and stored.

#### Individual Skills- and Scenario-Training Data (NNL Skills Training)

The newborn ventilation trainer, NNL (with an NNL application capable of recording and storing training data), was installed at all 30 hospitals. It provided an overview of the number of providers trained, training performance and progress, and areas of improvement. The training data captured from NNL (i.e., for both individual skills and scenario training, collectively termed NNL skills training) were uploaded automatically onto a central server. From the server, data were downloaded, checked for consistency by a data clerk, and transferred to statistical packages for analysis.

#### Mentorship and Supportive Supervision Data

**Figure 4.**
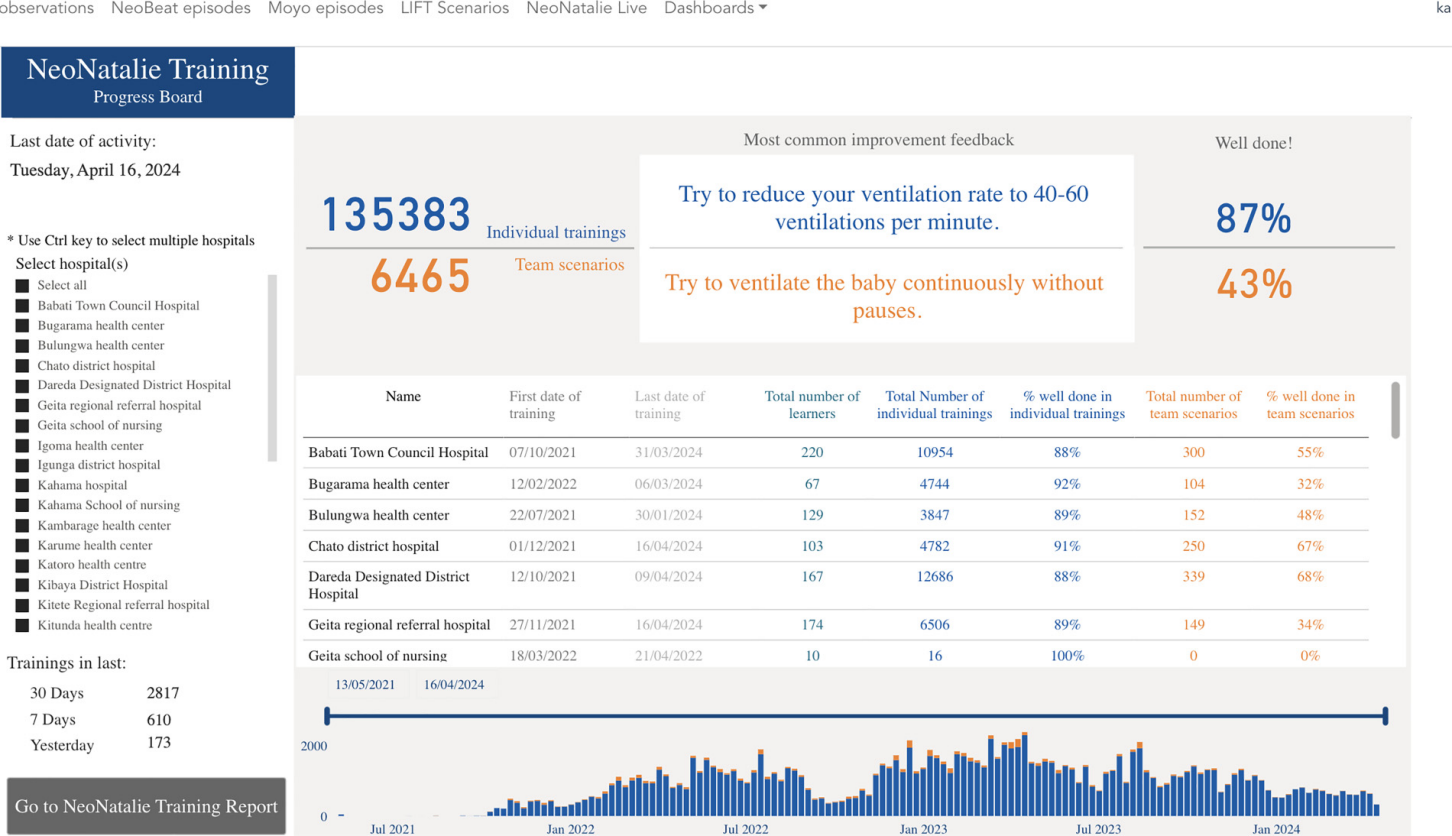
An excerpt from the NeoNatalie Live training progress board providing feedback to healthcare workers.

After each site visit, national facilitators would provide a report on different areas that they had observed, including practices using clinical and training tools, gaps identified, the causes of gaps, areas for improvement, interventions to be instituted, opportunities observed for improvement, and responsible personnel for each of the agreed action plans. The different training data sources are presented in Table 1, and an example of an NNL training progress board to be used for local CQI is illustrated in Figure 4.

#### Ethics Approval and Consent to Participate

This study was conducted in line with the Declaration of Helsinki and approved by the Institutional Review Board, the National Institute for Medical Research (Ref. NIMR/HQ/R.8a/Vol.IX/3458) in Tanzania (30 June 2020) and the Regional Ethical Committee (Ref. 229725) in Norway (August 6, 2021). The local IRB, the National Institute for Medical Research waived the need for HCWs and patient consent, as the study is a quality improvement project.

### Data Analysis

#### Quantitative

Descriptive analyses were used to show the distribution of training sessions, HCWs, and births. The tables display the counts, proportions, means, and standard deviations. The ratio of the average number of deliveries per HCW was calculated and used as a proxy for the burden/workload of HCWs in each facility. Scatter plots and correlation coefficients were used to show the relationship between the average training frequency and the average number of births per HCW for each region and facility level. The quantitative data were analyzed using R version 4.2.2 software (R Core Team, 2023).

#### Qualitative

A qualitative descriptive design was used to interpret the data. Open-ended responses in the supervision database were analyzed for thematic content using an inductive approach inspired by Graneheim and Lundman (2004). Two sections of the database were of particular interest. These were the *Strengths observed at this facility on this visit* and *Opportunities observed at this facility on this visit*, which were completed by the national facilitator after each visit. The analysis of the qualitative data focused on understanding the experiences of implementing the SBBC, gaining insights into the challenges and opportunities of implementing the intervention, and contextualizing the quantitative training data. After familiarization with the dataset by reading and rereading the text, the data were searched for repeated patterns of meaning relevant to the areas of interest. An initial set of descriptive codes was generated using NVIVO software. After deciding on a final set of codes, the codes were grouped into themes. All authors revised and agreed on the final set of codes and themes.

## Results

### Quantitative

A total of 1,290 HCWs were trained in the 30 chosen health facilities across the five regions. Initial training sessions were conducted in June 2021 (Manyara, *n* = 250), August 2021 (Tabora, *n* = 235), October 2021 (Geita, *n* = 248), January 2022 (Shinyanga, *n* = 255), and February 2022 (Mwanza, *n* = 302). Actual implementation at health facility levels started stepwise, as shown in Table 2, and the facility-level details are shown in the supplementary file. The regional monthly average number of facilitator-led *in-situ* team simulation training sessions ranged from six to eight. The monthly average number of individual skills- and scenario-training sessions using NNL (NNL skills training) ranged from 315 in Geita to 1,049 in Manyara. In most regions, there was a steady increase in NNL skills-training sessions, whereas facilitator-led *in-situ* team simulation training varied across months.

**Table 2.** The Average Number of Births per HCW per Month, by Region and Health Facility Levels.

	Region	Average number of births/months**	Estimated number of HCWs at a given time	Average number of births per HCW/month**
Regions	Overall	8,379 (498)	464	19 (7)
	Manyara	1,203 (89)	100	12 (1)
	Tabora	1,352 (232)	88	15 (3)
	Geita	2,358 (342)	76	31 (4)
	Shinyanga	1,433 (157)	90	16 (2)
	Mwanza	2,033 (241)	110	18 (2)
Facility levels	HC	2,599 (463)	142	18 (3)
	DH	4,435 (355)	228	19 (2)
	RRH	1,346 (158)	94	14 (2)

*Note.* DH = district hospitals; HC = health center; HCW = healthcare worker; RRH = regional referral hospitals.

**Numbers are presented in *mean (SD)*.

Table 2 presents the average number of births per HCW per month by region and facility levels, indicating the burden of HCWs. Overall, each HCW attended approximately 19 deliveries per month. The highest number was in Geita (*n* = 31), and the lowest was in Manyara (*n* = 12). Regional referral hospitals had the lowest rates of 14 deliveries per HCW.

**Figure 5.**
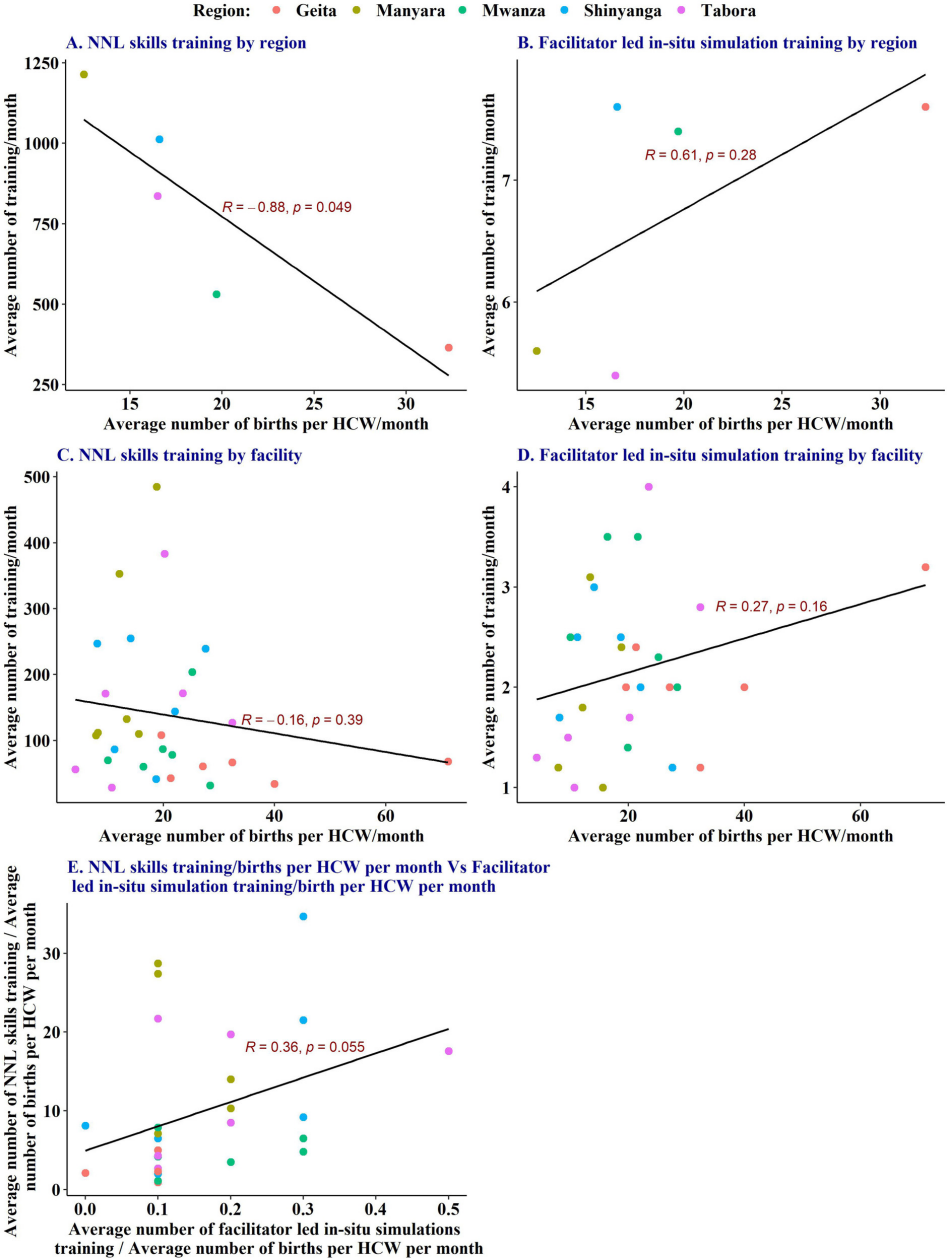
Average number of skills-training sessions using NeoNatalie Live and facilitator-led *in-situ* simulation training by facility.

**Figure 6.**
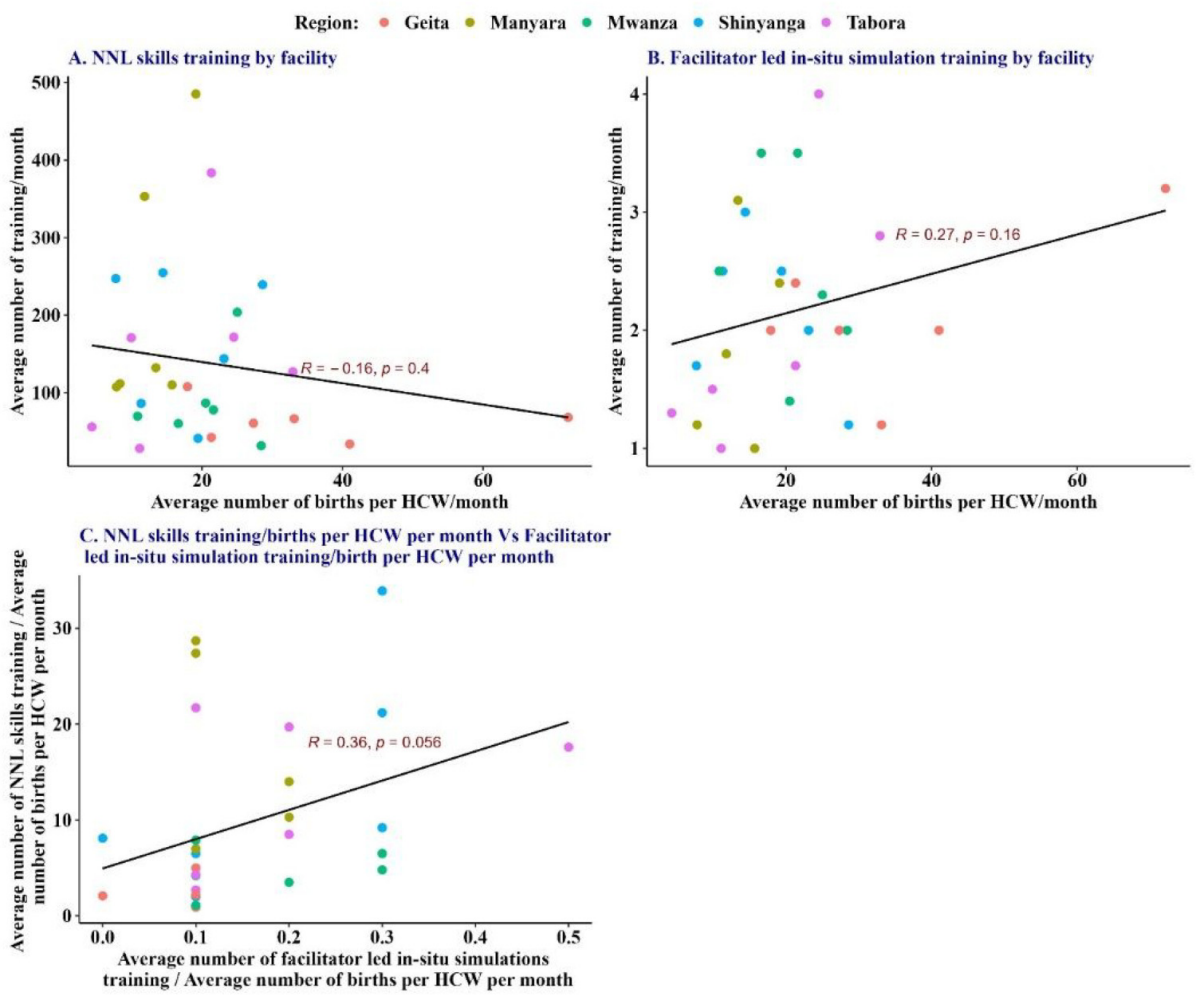
Average number of training sessions per month versus the average number of births per healthcare worker (HCW) per month, by region and facility.

Figure 5 illustrates the relationship between the average number of deliveries per HCW and the monthly training frequency at the health facility. At individual health facilities, there was no correlation between the clinical burden and an average number of NNL skills-training sessions (Figure 5a) or facilitator-led *in-situ* team simulation training (Figure 5b), which shows that simulation training took place irrespective of the HCWs’ workload. The details of this analysis by health facility and regions are attached as Appendix 1. Figure 6 shows a weak (*r* = 0.36) positive relationship between the frequency of NNL skills training per clinical burden and the frequency of facilitator-led simulations per clinical burden. However, this relationship was not statistically significant (*p* = .056). The relationship implies that in health facilities where NNL skills training took place, there was also a tendency for facilitator-led team simulation training to occur, although it was not statistically significant at .05.

### Qualitative Results

Qualitative analysis of the open-ended responses in the written reports completed by the national facilitators during monthly visits to the facilities identified the strengths and opportunities at each facility. The six facilities in one region, Tabora, did not have recorded data in the reports; these fields were left blank. Therefore, the qualitative dataset is based on the written reports from the 24 facilities in Geita, Manyara, Mwanza, and Shinyanga. Strengths and opportunities at these facilities included high motivation, full participation between stakeholders, and the active use of SBBC data.

#### The High Degree of Motivation and Participation among Stakeholders

A frequently reported strength was that all key stakeholders were described as motivated and participated in SBBC activities. This included hospital management, doctors in charge, and other HCWs: *Hospital management is motivated by SBBC activities and participates fully in them.* The motivation was also linked to positive views about the availability of SBBC clinical and training tools and that these were working well and being used. Many of the facilities had designated a training corner for SBBC training activities and ran *in-situ* simulations with numerous scenarios to mimic realistic clinical conditions. Facility champions were described as committed and ensured that whenever new staff joined the facility, they were also trained on SBBC: *Facility champions trained new nurses on how to use SBBC*. Healthcare workers were described as flexible and open to changing their ways of healthcare provision from business as usual to being more focused and data-driven. Facility champions showed flexibility and commitment to including everyone working in the labor ward in the implementation of the SBBC by also including those working only on the night shift, with reports stating *Flexibility*—*those on night duty participate in mentorship*. The national facilitators also noted that HCWs’ clinical skills were showing improvement following the training that they had received: *Have addressed gaps identified during the last supervision visit*.

#### Active Use of SBBC Data

Using and sharing locally collected facility/patient data to guide scenario training was frequently reported as strength. Here, it was stated that data were shared with all staff in the facility in different ways (such as by posting it on a notice board, sharing it in a WhatsApp group, or sharing it during weekly meetings) and that it was also shared with the administration, with reports stating *Data are visualized for staff to review or see easily*. *Data are being shared on WhatsApp groups.* The patient and training data collected as part of the SBBC was described as enhancing motivation. It was actively used to guide scenario training: *Data are displayed for all staff to read and review during weekly clinical meetings.* Staff also discussed missing data and gaps in documentation, and it was believed that the SBBC improved documentation. Although the overall feedback was positive from most of the facilities included, in one region it was recorded that: *They need more supervision and mentorship*. It should also be noted that the database does not indicate whether follow-up supervision occurred or other follow-up actions were implemented.

Although strengths and opportunities were reported for each facility in the four regions, the reports varied greatly in length and detail. This made it challenging to combine the qualitative and quantitative data at the facility level; therefore, trends are reported for the entire qualitative dataset instead. However, Tabora, the region that conducted the least facilitator-led *in-situ* team simulation training, is the region for which we lack qualitative data on strengths and opportunities.

## Discussion

Our data show a sustained high number of NNL skills training and facilitator-led team simulations by HCWs using SBBC tools after the initial facility training and the introduction of training tools. There were more individual NNL training sessions than facilitator-led team simulations, probably because these are relatively easy to execute. Despite a heavy clinical workload due to many monthly deliveries and a low number of HCWs, many reported simulation training sessions took place. However, some health facilities only conducted a few simulation training sessions during the study period. The facilitator-led *in-situ* simulation training sessions require more logistics, are planned, and may be executed despite a high clinical burden. The high number of reported training sessions corresponds with the qualitative data, which found a high degree of motivation, full participation by all stakeholders, and the active use of data at facilities in four of the five regions included. The region, which lacked qualitative data about these enabling factors, also conducted the fewest facilitator-led team simulations.

The findings that both NNL skills training and facilitator-led *in-situ* simulation training were sustained throughout all levels of facilities (i.e., HC, DH, and RRH) may have contributed to the observed trends of increased newborn and maternal survival reported in the halfway evaluation (Ersdal et al., 2023). This implies that simulations, both individual and facilitator-led simulation training, are essential as intermediary steps toward the reduction of newborn and maternal mortality. Several factors could explain the sustained simulation training. The SBBC emphasized ownership, and the implementation process of the whole bundle included all stakeholders, which was confirmed by the qualitative data. A previous qualitative study found a high acceptance of the SBBC tools and the bundle among HCWs (Mdoe et al., 2023). Having facility champions in each facility as motivators for continuous practice and the involvement of regional health management teams during supportive supervision visits to oversee CQI ensured a high degree of local ownership and buy-in from key stakeholders. This ensured that HCWs were supported and encouraged to practice (Ugwa et al., 2020). Vadla et al. reported similar findings of sustained training practice in the presence of motivators in a similar setting (Vadla et al., 2022a. Facilitator-led *in-situ* simulation training was encouraged and practiced during all mentorship visits, and in time, HCWs could use designated scenarios, resulting in frequent facilitator-led *in-situ* simulation training. The study findings concur with Chang et al. (2022), who state that simulation-based training is feasible and sustainable in LMICs.

Nevertheless, *in-situ* facilitator-led team simulations may be challenging to organize and conduct in busy facilities. Thus, we registered a much lower number of such training sessions than shorter and independently performed NNL training sessions. Additionally, some facilities carried out fewer simulation training sessions throughout the study period than others. Tailored mentorship to these sites to identify and solve challenges may be crucial to bringing them to the desired level.

The improved high-fidelity NNL simulator for independent training, capable of providing automatic and objective performance feedback to the provider, was installed in the labor wards of all the SBBC sites. This feedback likely contributed to the increased frequency of individual skills training, as it motivates HCWs to monitor their ventilation performance (Vadla et al., 2022a). NeoNatalie Live allows individuals or groups to monitor and follow their performance over time. Previous studies from both high- and low-income settings found that using NNL improves and maintains ventilation skills (Haynes et al., 2021; Vadla et al., 2022a). Combined with facilitator-led *in-situ* team simulation training, NNL skills training has improved training performance and newborn ventilation skills and reduced the time from birth to first ventilation when used in Tanzania (Vadla et al., 2022b). Additionally, *in-situ* simulation offered an opportunity for HCWs to continue to learn without needing to leave their facility, which could otherwise have increased the workload of those remaining. For simulation training efforts to be effective, issues related to adequate human resources for health must be addressed (Seethamraju et al., 2022).

Previous Helping Babies Breathe programs linked to CQI initiatives had shown sustained gains, primarily due to skills maintenance (Rule et al., 2017). The current study shows prolonged LDHF practices are necessary to build a culture of clinical practice in safe environments using simulations (Mduma et al., 2015a; Mdoe et al., 2023). Such a culture has already been cultivated in some SBBC facilities, as evidenced by the findings that those facilities that conduct more NNL skills training are more likely to conduct facilitator-led team simulations (Ersdal et al., 2023).

The results of the present study show that motivation and participation were considered important factors, which also partly explains the high level of sustained individual/independent and facilitator-led simulation training. Several factors could have contributed to this sustained motivation throughout the implementation period. Gaining new knowledge and skills through training can enhance HCWs’ feeling of empowerment and thereby stimulate greater participation (Bruce et al., 2019). The fact that hospital management and supervisors also participated and were positive toward SBBC might have enhanced the empowerment among the HCWs because they experienced their newly acquired skills being valued. This is in line with a previous study from Mozambique, where graduate nurses felt powerless and perceived barriers because of a lack of management engagement and active participation in the implementation processes at the workplace (Bruce et al., 2019). Previous findings indicate that support from senior staff and ownership by opinion leaders is key to the successful implementation of interventions in low-resource settings (Torloni et al., 2023).

Maintaining motivation and participation to learn and acquire new skills is crucial to maintaining a safe clinical learning environment that is conducive to learning. Developing and maintaining a positive clinical learning environment depends on several factors, such as the attitude and skills of the supervisor, the quality of the feedback received, and the context (Dale et al., 2013; Gonekani Mwale & Kalawa, 2016). To ensure that skills-training enhances performance, the supervisor's competency is an essential success factor. A qualitative study found that SBBC fostered a “no-blame culture” by emphasizing the importance of learning from mistakes through debriefing and supportive supervision (Mdoe et al., 2023). Previous findings indicate various challenges when mentoring HCWs in low-resource settings, such as a lack of time, support, and teamwork (Mhango et al., 2021). A key component of the SBBC was the formal training in simulation pedagogics in the form of the SimBegin training program, in which the national facilitators participated and were certified. The training program included sessions on the importance of creating a psychologically safe and positive learning environment, and of supportive supervision and mentorship. The study findings suggest that these skills were adopted by the facility champions being trained by the national facilitators, which enabled them to create an environment that was conducive to learning and behavior change at the health facilities. Capacity building of national facilitators at the center of the SBBC through SimBegin training and the subsequent cascade of SBBC training, together with regular mentorship and supportive supervision visits, might have been a key to its success as the findings indicate both high levels of motivation and consistency in carrying out regular training sessions among participants.

### Strengths and Limitations

The mixed methods approach provides a comprehensive evaluation of the implementation of *in-situ* LDHF simulation-based training and the experiences of using the SBBC bundle among HCWs and stakeholders in Tanzania. Integrating quantitative data with qualitative insights enabled a more holistic understanding of the bundle's effectiveness and the factors influencing its uptake. This approach enhanced the validity of the study findings by capturing both objective measures and subjective experiences, thereby informing tailored strategies for successful implementation. However, we could not link simulation practice with clinical outcomes, which will be analyzed and included in other forthcoming publications. Also, the qualitative dataset does not include the strengths and opportunities for the six facilities in the Tabora region, as this information was not included in the written reports. The qualitative data would have been richer if it had also included other qualitative data collection methods, such as individual semistructured interviews or focus group discussions with participants.

## Implications for Practice

The study highlights significant implications for the practice of nurses and midwives by demonstrating that integrating simulation-based training into routine practices at all healthcare facility levels can significantly enhance maternal and newborn outcomes. This suggests that there is a need for nurses and midwives to engage in sustained training efforts, emphasizing both individual skill development and facilitator-led team simulations. Advanced simulation equipment provides valuable feedback and may support ongoing skills enhancement and competency maintenance. Creating a safe clinical learning environment through supportive supervision and debriefing sessions should be emphasized. Moreover, formal training programs for national facilitators are essential for sustaining and expanding these training efforts, highlighting the importance of ongoing professional development in nursing and midwifery practice.

## Conclusions and Recommendations

Ensuring that HCWs are skilled in managing complications during and after birth is key to reducing newborn and maternal mortality in LMICs. The study findings show a high degree of motivation to build emergency obstetric and perinatal care skills through *in-situ* LDHF training, mentorship, supportive supervision, and the active use of real-time facility data among HCWs and stakeholders at SBBC facilities in Tanzania. The findings also indicate that facilitator-led *in-situ* simulation training was more likely to take place where individual skills-training sessions were recorded. Additionally, the number of reported training sessions took place regardless of an increased workload due to the high number of monthly deliveries and the low number of HCWs. Tailored mentorship is recommended for sites with low performance.

An inclusive approach that included local health authorities, different cadres working together in the labor ward, and senior management at the health facilities, combined with mentorship and supportive supervision, was beneficial in creating an environment that was conducive to learning and CQI. Simulation training requires allocating time, personnel, and facilitators to make it a more structured form of training and easier for HCWs to commit to.

## Supplemental Material

sj-docx-1-son-10.1177_23779608241309447 - Supplemental material for Practice, Experiences, and Facilitators of Simulation-Based Training During One Year of Implementation in 30 Hospitals in Tanzania Supplemental material, sj-docx-1-son-10.1177_23779608241309447 for Practice, Experiences, and Facilitators of Simulation-Based Training During One Year of Implementation in 30 Hospitals in Tanzania by Benjamin A. Kamala, Robert Moshiro, Florence S. Kalabamu, Torgeirsen Kjetil, Godfrey Guga, Beatrice Githiri, Justine Samson, Philimon Chavala, Grace Qorro, Damas Kayera, Ivony Kamala, Catherine Massay, Paschal Mdoe, Vickfarajaeli Daudi, Esto Mduma, Shally Mwashemele, Felix Bundala, Hege Ersdal and Sara Rivenes Lafontan in SAGE Open Nursing
