# Supplementary material for: Practice, Experiences, and Facilitators of Simulation-Based Training During One Year of Implementation in 30 Hospitals in Tanzania
Source: SAGE Open Nurs. 2025 Jan 3;11:23779608241309447. doi: 10.1177/23779608241309447 (PMC11696965; doi:10.1177/23779608241309447)
Supplement: sj-docx-1-son-10.1177_23779608241309447 - Supplemental material for Practice, Experiences, and Facilitators of Simulation-Based Training During One Year of Implementation in 30 Hospitals in Tanzania [file sj-docx-1-son-10.1177_23779608241309447.docx]

| **Month** | **Jul-21** | **Aug-21** | **Sep-21** | **Oct-21** | **Nov-21** | **Dec-21** | **Jan-22** | **Feb-22** | **Mar-22** | **Apr-22** | **May-22** | **Jun-22** | **Jul-22** | **TOTAL** | **Monthly Average** | **Median no. training** |  |
| --- | --- | --- | --- | --- | --- | --- | --- | --- | --- | --- | --- | --- | --- | --- | --- | --- | --- |
| **NNL skills training** |  |  |  |  |  |  |  |  |  |  |  |  |  |  |  |  |  |
| Babati Hosp |  |  |  | 169 | 337 | 309 | 689 | 484 | 698 | 632 | 755 | 476 | 302 | **4,851** | **485** | **480** |  |
| Dareda Ddh |  |  |  | 301 | 160 | 153 | 72 | 302 | 499 | 535 | 549 | 595 | 365 | **3,531** | **353** | **334** |  |
| Kibaya Hosp |  |  | 10 | 14 | 99 | 17 | 40 | 46 | 237 | 479 | 119 | 101 | 68 | **1,230** | **112** | **68** |  |
| Mbulu Hosp |  |  | 5 | 19 | 99 | 98 | 71 | 61 | 364 | 91 | 138 | 114 | 151 | **1,211** | **110** | **98** |  |
| Mirerani Hc |  |  |  | 10 | 27 | 8 | 82 | 61 | 144 | 155 | 254 | 187 | 148 | **1,076** | **108** | **113** |  |
| Tumaini Hosp |  |  | 5 | 7 | 34 | 20 | 95 | 66 | 251 | 237 | 268 | 229 | 243 | **1,455** | **132** | **95** |  |
| Igunga hosp | - | - | - | 10 | 74 | 2 | 10 | 258 | 161 | 111 | 207 | 178 | 259 | **1,270** | **127** | **136** |  |
| Kitete RRH | - | - | - | 0 | 29 | 30 | 59 | 58 | 102 | 180 | 556 | 223 | 301 | **1,538** | **154** | **81** |  |
| Kitunda Hc | - |  | 10 | 0 | 36 | 76 | 34 | 3 | 23 | 20 | 20 | 33 | 28 | **283** | **26** | **23** |  |
| Nzega hosp | - | - | - | 4 | 135 | 68 | 49 | 87 | 181 | 202 | 308 | 351 | 332 | **1,717** | **172** | **158** |  |
| Upuge Hc | - | - | - | 10 | 71 | 30 | 52 | 42 | 41 | 24 | 89 | 103 | 98 | **560** | **56** | **47** |  |
| Urambo DH | - | - | - | 79 | 452 | 351 | 408 | 385 | 371 | 343 | 527 | 553 | 366 | **3,835** | **384** | **378** |  |
| Chato DH | - | - | - | - | - | 50 | 20 | 18 | 23 | 26 | 53 | 94 | 56 | **340** | **43** | **38** |  |
| Geita RRH | - | - | - | - | 10 | 108 | 111 | 34 | 80 | 97 | 217 | 190 | 124 | **971** | **108** | **108** |  |
| Katoro Hc | - | - | - | - | 118 | 77 | 31 | 3 | 18 | 17 | 119 | 74 | 157 | **614** | **68** | **74** |  |
| Masumbwe Hc | - | - | - | - | 10 | 48 | 0 | 28 | 11 | 24 | 76 | 37 | 37 | **271** | **30** | **33** |  |
| Nzera hosp | - | - | - | - | 9 | 34 | 32 | 0 | 9 | 50 | 120 | 163 | 69 | **486** | **54** | **42** |  |
| Uyovu Hc | - | - | - | - | 11 | 120 | 70 | 77 | 87 | 101 | 77 | 45 | 11 | **599** | **67** | **77** |  |
| Bugarama Hc | - | - | - | - | - | - | - | 112 | 119 | 77 | 57 | 105 | 48 | **518** | **86** | **91** |  |
| Bulungwa Hc | - | - | - | - | - | - | - | 156 | 142 | 74 | 467 | 424 | 266 | **1,529** | **255** | **211** |  |
| Kahama hosp | - | - | - | - | - | - | - | 175 | 152 | 147 | 205 | 271 | 486 | **1,436** | **239** | **190** |  |
| Kambarage Hc | - | - | - | - | - | - | - | 111 | 183 | 49 | 127 | 95 | 299 | **864** | **144** | **119** |  |
| Nindo Hc | - | - | - | - | - | - | - | 26 | 25 | 25 | 89 | 51 | 32 | **248** | **41** | **29** |  |
| Shinyanga RRH | - | - | - | - | - | - | - | 240 | 185 | 134 | 175 | 411 | 338 | **1,483** | **247** | **213** |  |
| Igoma HC | - | - | - | - | - | - | - | 158 | 99 | 69 | 73 | 60 | 62 | **521** | **87** | **71** |  |
| Karume HC | - | - | - | - | - | - | - | 84 | 123 | 68 | 55 | 40 | 49 | **419** | **70** | **62** |  |
| Nansio DH | - | - | - | - | - | - | - | 18 | 80 | 12 | 11 | 2 | 68 | **191** | **32** | **15** |  |
| Nyamagana DH | - | - | - | - | - | - | - | 93 | 132 | 291 | 158 | 160 | 389 | **1,223** | **204** | **159** |  |
| Sekou Tourre RRH | - | - | - | - | - | - | - | 43 | 103 | 98 | 85 | 50 | 89 | **468** | **78** | **87** |  |
| Sengerema DDH | - | - | - | - | - | - | - | 71 | 36 | 20 | 95 | 60 | 80 | **362** | **60** | **66** |  |
| **Facilitator-led team simulation training** | | | | | | | | | | | | | | | | | |
| Babati Hosp | - | - | - | - | - | 2 | 1 | 2 | 3 | 0 | 2 | 4 | 3 | **17** | **2** | **3** |  |
| Dareda Ddh | 2 | 0 | 5 | 3 | 3 | 1 | 1 | 1 | 1 | 1 | 1 | 1 | 0 | **20** | **2** | **1** |  |
| Kibaya Hosp | - | - | - | - | - | - | 0 | 0 | 0 | 0 | 0 | 0 | 0 | **0** | **0** | **0** |  |
| Mbulu Hosp | 1 | - | - | - | - | - | 0 | 0 | 0 | 0 | 0 | 1 | 1 | **3** | **1** | **0** |  |
| Mirerani Hc | - | - | - | - | - | - | 1 | 1 | 0 | 2 | 1 | 0 | 0 | **5** | **1** | **1** |  |
| Tumaini Hosp | - | - | - | - | - | - | 2 | 2 | 2 | 4 | 5 | 3 | 4 | **22** | **3** | **3** |  |
| Igunga hosp | - | - | - | - | - | - | 0 | 1 | 0 | 0 | 4 | 3 | 3 | **11** | **2** | **2** |  |
| Kitete RRH | - | - | - | - | - | - | 0 | 0 | 0 | 0 | 1 | 2 | 0 | **3** | **1** | **0** |  |
| Kitunda Hc | - | - | - | - | - | - | 1 | 0 | 0 | 2 | 0 | 0 | 0 | **3** | **1** | **0** |  |
| Nzega hosp | - | - | - | - | - | - | 0 | 0 | 0 | 0 | 3 | 0 | 5 | **8** | **2** | **0** |  |
| Upuge Hc | - | - | - | - | - | - | 0 | 0 | 0 | 0 | 2 | 1 | 1 | **4** | **1** | **1** |  |
| Urambo DH | - | - | - | - | - | - | 1 | 0 | 0 | 1 | 1 | 2 | 4 | **9** | **2** | **1** |  |
| Chato DH | - | - | - | - | - | - | 2 | 2 | 3 | 3 | 2 | 0 | 0 | **12** | **2** | **2** |  |
| Geita RRH | - | - | - | - | - | - | 1 | 0 | 1 | 1 | 5 | 0 | 0 | **8** | **1** | **1** |  |
| Katoro Hc | - | - | - | - | - | - | 2 | 11 | 4 | 1 | 1 | 4 | 2 | **25** | **4** | **3** |  |
| Masumbwe Hc | - | - | - | - | - | - | 0 | 0 | 0 | 2 | 2 | 0 | 0 | **4** | **1** | **0** |  |
| Nzera hosp | - | - | - | - | - | - | 0 | 2 | 0 | 1 | 0 | 3 | 0 | **6** | **1** | **1** |  |
| Uyovu Hc | - | - | - | - | - | - | 1 | 1 | 0 | 0 | 1 | 2 | 0 | **5** | **1** | **1** |  |
| Bugarama Hc | - | - | - | - | - | - | 1 | 0 | 7 | 1 | 0 | 1 | 0 | **10** | **1** | **1** |  |
| Bulungwa Hc | - | - | - | - | - | - | 0 | 2 | 3 | 2 | 0 | 5 | 0 | **12** | **2** | **2** |  |
| Kahama hosp | - | - | - | - | - | - | 0 | 1 | 1 | 0 | 1 | 2 | 1 | **6** | **1** | **1** |  |
| Kambarage Hc | - | - | - | - | - | - | 0 | 0 | 0 | 3 | 2 | 1 | 1 | **7** | **1** | **1** |  |
| Nindo Hc | - | - | - | - | - | - | 0 | 1 | 4 | 3 | 0 | 2 | 0 | **10** | **1** | **1** |  |
| Shinyanga RRH | - | - | - | - | - | - | 0 | 0 | 1 | 0 | 0 | 2 | 0 | **3** | **1** | **0** |  |
| Igoma HC | - | - | - | - | - | - | 0 | 0 | 1 | 1 | 1 | 2 | 2 | **7** | **1** | **1** |  |
| Karume HC | - | - | - | - | - | - | 0 | 7 | 0 | 1 | 1 | 1 | 0 | **10** | **1** | **1** |  |
| Nansio DH | - | - | - | - | - | - | 1 | 2 | 4 | 4 | 1 | 1 | 1 | **14** | **2** | **1** |  |
| Nyamagana DH | - | - | - | - | - | - | 1 | 0 | 0 | 0 | 5 | 1 | 0 | **7** | **1** | **0** |  |
| Sekou Tourre RRH | - | - | - | - | - | - | 3 | 0 | 0 | 0 | 0 | 0 | 4 | **7** | **1** | **0** |  |
| Sengerema DDH | - | - | - | - | - | - | 0 | 2 | 0 | 0 | 5 | 0 | 0 | **7** | **1** | **0** |  |
